# Supplementary material for: Methotrexate attenuates vascular inflammation through an adenosine-microRNA-dependent pathway
Source: eLife. 2021 Jan 8;10:e58064. doi: 10.7554/eLife.58064 (PMC7840179; doi:10.7554/eLife.58064)
Supplement: Supplementary file 1. [file elife-58064-supp1.pdf]

Supplementary File 1. *MiR-181a2b2* promoter sequences

MiR-181a2b2 truncated (606bp) promoter sequence:

GCCACTTTTATTTTTACCTTATCAAACCTATAAAATCAAGACCAAGGGTTGGTCTGGATATGAAGCTTGGTTAGGA
TCACAATAAATTTTCAAATCAAATAATTGGTTTCAGTTAGAGCCAAGTTTATCAACAATAGGGTGATTTAGGATTCA
GGAAAAATCGGCTGCCTATTTGTATTTCAGAAAGACTAGTATTTCTTCTGACACAAGTAGAGAGGTGTAAGCAAC
CTCTGGGTGGGTAATGCCACCAAACAAAAGCAACACCTTCAAAGTCTCTCAGTGACTAGAGGCAGCCAGACACT
CAAACTGCCAGTCCTGAATTACGGCCTCTGAATGGTGAGGGCTTCACATTTCACCGATCAGGGCACAGCCCAAT
CAGAAGACAAACCCCTGCACTGCCAGGGACCAGCAAATCCGCTTTCTTCCAGTGTGCACATGACTAAATGTGCT
TCTGTCCATCATCTGGGTCATATATAGTTCAATGCGAGCTGAGCAGACAGGGCTGCAAGGAAATCTGGCGCGGT
TCAATACCTCGTCTAGCCTGGGTTCCAGTATCTAATTTTTTTTTTGTTTTAACTGACAAACTCATTTCTCTACTGGG
ACAGG

MiR-181a2b2 truncated (402bp) promoter sequence:

CACAAGTAGAGAGGTGTAAGCAACCTCTGGGTGGGTAATGCCACCAAACAAAAGCAACACCTTCAAAGTCTCTC
AGTGACTAGAGGCAGCCAGACACTCAAACTGCCAGTCCTGAATTACGGCCTCTGAATGGTGAGGGCTTCACATT
TCACCGATCAGGGCACAGCCCAATCAGAAGACAAACCCCTGCACTGCCAGGGACCAGCAAATCCGCTTTCTTCC
AGTGTGCACATGACTAAATGTGCTTCTGTCCATCATCTGGGTCATATATAGTTCAATGCGAGCTGAGCAGACAGG
GCTGCAAGGAAATCTGGCGCGGTTCAATACCTCGTCTAGCCTGGGTTCCAGTATCTAATTTTTTTTTTGTTTTAAC
TGACAAACTCATTTCTCTACTGGGACAGG

MiR-181a2b2 truncated (301bp) promoter sequence:

ACTGCCAGTCCTGAATTACGGCCTCTGAATGGTGAGGGCTTCACATTTCACCGATCAGGGCACAGCCCAATCAG
AAGACAAACCCCTGCACTGCCAGGGACCAGCAAATCCGCTTTCTTCCAGTGTGCACATGACTAAATGTGCTTCT
GTCCATCATCTGGGTCATATATAGTTCAATGCGAGCTGAGCAGACAGGGCTGCAAGGAAATCTGGCGCGGTTCA
ATACCTCGTCTAGCCTGGGTTCCAGTATCTAATTTTTTTTTTGTTTTAACTGACAAACTCATTTCTCTACTGGGACA
GG

MiR-181a2b2 truncated (150bp) promoter sequence:

CATCATCTGGGTCATATATAGTTCAATGCGAGCTGAGCAGACAGGGCTGCAAGGAAATCTGGCGCGGTTCAATA
CCTCGTCTAGCCTGGGTTCCAGTATCTAATTTTTTTTTTGTTTTAACTGACAAACTCATTTCTCTACTGGGACAGG
