# Supplementary material for: Methotrexate attenuates vascular inflammation through an adenosine-microRNA-dependent pathway
Source: eLife. 2021 Jan 8;10:e58064. doi: 10.7554/eLife.58064 (PMC7840179; doi:10.7554/eLife.58064)
Supplement: Supplementary file 2. [file elife-58064-supp2.pdf]

Supplementary File 2. Primer list

Gene name	Forward	*Reverse*
Human-*ADORA1*	CAAGATCCCTCTCCGGTACA	CAGCCAAACATAGGGGTCAG
Human-ADORA2A	CTGGCTGCCCCTACACATC	TCACAACCGAATTGGTGTGGG
Human-*ADORA2B*	TGCACTGACTTCTACGGCTG	GGTCCCCGTGACCAAACTT
Human-*ADORA3*	GGCCAATGTTACCTACATCACC	CCAGGGCTAGAGAGACAATGAA
Human-*SMAD2*	CGTCCATCTTGCCATTCACG	CTCAAGCTCATCTAATCGTCCTG
Human-*SMAD3*	TGGACGCAGGTTCTCCAAAC	CCGGCTCGCAGTAGGTAAC
Human-*SMAD4*	CTCATGTGATCTATGCCCGTC	AGGTGATACAACTCGTTCGTAGT
Human-*VCAM-1*	CCCACAGTAAGGCAGGCTGT	GCTGGAACAGGTCATGGTCA
Human-*ICAM-1*	CGCACTCCTGGTCCTGCT	AACAACTTGGGCTGGTCACA
Human-*E-Selectin*	GGGCATGTGGAATGATGAGA	CACTGAAGCCAGGGTCACAC
Human-*HPRT*	GCTATAAATTCTTTGCTGACCTG CTG	AATTACTTTTATGTCCCCTGTTGAC TGG
Mouse-*Vcam-1*	CAACATGTGGCTCTGGGAAG	GCCAAACACTTGACCGTGAC
Mouse- *β-actin*	GAAATCGTGCGTGACATCAAAG	TGTAGTTTCATGGATGCCACAG
